# The outcomes of the obesity paradox in pulmonary embolism: a study of the national inpatient sample database from 2016 to 2020

**DOI:** 10.1007/s00277-025-06197-1

**Published:** 2025-01-22

**Authors:** Ayushi Garg, Aman Saleemi, Mekdes Asfaw, Nour Aldaoud, Pranav Chalasani, Vamsi Krishna Lavu, Palpasa Bhui, Tripti Nagar, Ankit Agarwal, Abdullah Yesilyaprak, Jai Kumar, Mohamed Mansour, David Bock, Joiven Nyongbella, Zain Kulairi

**Affiliations:** 1 Internal Medicine- Wayne State University School of Medicine, Rochester Hills, Michigan, USA; 2 Internal Medicine- Cleveland Clinic Foundation, Cleveland City, Ohio USA

**Keywords:** Obesity, Pulmonary embolism, Paradox, Case report

## Abstract

The “obesity paradox” suggests that, despite a higher baseline risk for adverse health outcomes, obese patients can experience a lower complication and mortality rate in conditions such as pulmonary embolisms (PE). This study aims to examine the association between obesity and inpatient outcomes of PE patients, utilizing the data from the National Inpatient Sample (NIS) database. We conducted a retrospective study analysis of obese adult PE patients (aged ≥ 18) using the NIS database from 2016 to 2020. Patients were categorized as either obese (body mass index (BMI) ≥ 30) or non-obese, excluding those with cancer diagnosis and age > 75 years to reduce confounding factors. Multivariable logistic regression, adjusted for confounders, compared the inpatient outcomes, including mortality, length of stay, need for mechanical invasive ventilation (MIV), incidence of shock requiring vasopressor use and use of reperfusion therapies. Our results showed obese patients had a lower in-hospital mortality and reduced risk for certain adverse outcomes when compared to non-obese patients. Limitations in our data, such as the lack of imaging confirmation and inability to track certain risk indicators in real time, affected precision in outcome severity classification. Our findings support the existence of an obesity paradox, particularly in PE patients, with obese patients experiencing better inpatient outcomes relative to their non-obese counterparts. This study advances the understanding of obesity’s complex role in PE outcomes. However, further research is needed to further elucidate potential protective mechanisms to address our study limitations.

## Introduction

Obesity has been on the rise globally, with approximately 890 million adults 18 years of age and older being classified as obese, as per the World Health Organization data [1]. In adults, overweight is defined as a body mass index (BMI) of 25 to 29.9 kg/m2 and obesity as BMI ≥ 30 kg/m² [2]. In the US, obesity has reached epidemic levels and is associated with an increased incidence and severity of numerous complications including stroke, malignancies, cardiovascular diseases including coronary artery disease, heart failure, atrial fibrillation, venous thromboembolism and pulmonary embolism (PE), collectively contributing to increased morbidity and mortality rates [3–5].

Pulmonary embolism, a potentially fatal condition characterized by obstruction of the pulmonary artery, affects nearly 370,000 patients per year in the US alone [1]. The relationship between obesity and outcomes in PE patients remains an area of active research with some studies reporting improved survival rates amongst obese patients, while others find no significant survival benefit [6–8]. This study aims to clarify the impact of obesity on adverse in-hospital outcomes in patients diagnosed with PE.

The term ‘obesity paradox’ describes the observation that obese patients can often experience more favorable outcomes, including lower complication and mortality rates, in conditions such as heart failure [9], PE [10] and chronic kidney disease [11], despite a higher baseline risk of developing serious comorbidities compared to nonobese patients [6]. This paradoxical phenomenon suggests a potential protective mechanism associated with obesity, especially in patients with PE, although the underlying reason for this effect is poorly understood.

The relationship between obesity and mortality exhibits a U-shaped curve where both low and high BMI levels are associated with increased mortality risk, while moderate BMI levels may correlate with reduced mortality. Low BMI is linked with malnutrition, muscle wasting or chronic disease whereas high BMI is linked to metabolic dysregulation and chronic inflammatory conditions. This nuanced association underscores that varying degrees of obesity carry distinct mortality risk. Mild to moderate obesity may be linked with a neutral or slightly reduced mortality risk, a phenomenon termed as the “obesity paradox”, particularly observed in specific populations, such as older adults or those with chronic illnesses. In contrast, severe obesity (BMI > 35) consistently demonstrates a strong association to elevated mortality, driven largely by comorbidities such as cardiovascular disease and type 2 diabetes. In addition, the use of BMI as a parameter of obesity is also controversial, as it does not differentiate between lean mass and fat mass or capture fat distribution, which may affect health outcomes [12].

This study utilized the National Inpatient Sample (NIS) database, which contains data from 20% of inpatient hospitalizations across the participating states [13]. We identified adult patients diagnosed with PE from 2016 to 2020, categorizing them into obese (BMI ≥ 30 kg/m²) and non-obese groups. In-hospital outcomes, including comorbidities, use of reperfusion therapy and mortality, were compared between the two groups. Adjustments were made for potential confounders such as age, gender, coronary artery disease, congestive heart failure, atrial fibrillation/flutter, peripheral vascular disease, hypertension, type 2 diabetes mellitus, chronic kidney disease, chronic obstructive pulmonary disease, liver disease, rheumatological disease, history of deep vein thrombosis to further clarify the association between obesity and PE outcomes.

This study builds on a previous analysis of the NIS database from 2002 to 2014, which reported an increased risk of complications and poorer outcomes in non-obese PE patients compared to their obese counterparts [6]. By using more recent data (2016–2020), this nationwide study aimed to provide updated insights into complication rates and mortality outcomes among obese vs. nonobese PE patients.

## Materials and methods

### Data source

The National Inpatient Sample (NIS) database is the largest inpatient healthcare database in the US, comprising data from approximately 20% of hospitalizations across states participating in the Healthcare Cost and Utilization Project. It includes information from over 7 million hospitalizations annually, representing more than 97% of the US population [13]. Patient confidentiality is maintained, as the database excludes patient, hospital, and state identifiers. Because the NIS provides publicly accessible and de-identified data, studies utilizing this dataset do not require Institutional Review Board (IRB) approval.

### Study population

This retrospective cross-sectional study utilized the NIS database from 2016 to 2020, focusing on adult patients (aged 18 years and above) with a discharge diagnosis of PE, identified by ICD-10 code I26. Although diagnostic confirmation through imaging was not available within the NIS dataset, we assume clinical coding standards likely align these diagnoses with confirmatory testing with either computed tomography with pulmonary angiography (CTPA) or ventilation-perfusion (V/Q) scan results.

Obese patients with a BMI ≥ 30 kg/m2 were identified using the Elixhauser comorbidity measure within the NIS database and compared to non-obese patients to assess differences in adverse in-hospital outcomes. Morbid obesity categories (BMI > 40 and > 50) were not differentiated. Patients with a known cancer diagnosis and those older than 75 years of age were excluded from this study to focus the analysis on obesity-related outcomes, minimizing the influence of comorbidities that independently contribute to PE risk.

Data variables collected for analysis included demographic characteristics hospital size and teaching status, and relevant clinical comorbidities (Table 1). We also compared the utilization of reperfusion therapies, including thrombolysis, thrombectomy or embolectomy. The primary outcomes of interest were in-hospital mortality, length of stay in days, need for MIV and shock requiring vasopressor support. Reperfusion therapies, including thrombolysis, thrombectomy, or embolectomy were also analyzed.

This data was analyzed using descriptive statistics and reported as mean ± SD for continuous variables and percentages for categorical variables. The χ2 test or Fisher exact test was used as appropriate for categorical variables. A multivariable logistic regression model adjusted for age, gender, race, comorbidities and treatment/interventions was performed.

All analyses were conducted using the StataCorp 2021 (Stata Statistical Software: Release 17; StataCorp LLC, College Station, TX), with appropriate weights provided by the Healthcare Cost and Utilization Project to generate national estimates.

## Results

### Study population

A total of 1,940,294 PE-related discharges for patients aged ≥ 18 years were identified from the NIS database from 2016 to 2020, of which 436,505 patients (22%) classified as obese. Obese PE patients were slightly younger (mean age 58.4 ± 15.1 years) compared to their non-obese counterparts (mean age 64.0 ± 17.1 years, *p* < 0.001). The proportion of females was higher in the obese group (57.4%) compared to non-obese group (49.1%, *p* < 0.001). Ethnicity data revealed that White patients comprised 68.7% of obese PE patients and 70.9% of non-obese PE patients, while Black patients accounted for 17.5% of obese PE patients and 21.2% of non-obese PE patients (*p* < 0.001). In terms of insurance coverage, both obese and non-obese PE patients were more likely to be insured by Medicare (44.2% and 32.9%, respectively, *p* < 0.001) and less likely to have private insurance (32.9% and 24.4%, respectively, *p* < 0.001).

Regarding comorbid conditions, rates of congestive heart failure, hypertension, type 2 diabetes mellitus and renal failure were higher in obese patient cohort. The difference in the prevalence of hypertension was most significant with 70.6% of obese PE patients having comorbid hypertension, whereas only 21.2% of non-obese patients had hypertension. The p-values for all the above listed comorbidities demonstrated statistically significant with *p* < 0.001. Detailed baseline characteristics including demographic data, insurance coverage, hospital demographic information and comorbidities are summarized in Table 1.

**Table 1:** Baseline characteristics of pulmonary embolism patients with and without obesity (*table view*)

Characteristics [Mean(SD)] OR [*N*(%)]	Total (*n* = 1,940,294)	Not Obese (*n* = 1,503,789)	Obese (*n* = 436,505)	*P*-value
**Demographics**				
Age (years)	62.7 ± 16.9	64.0 ± 17.1	58.4 ± 15.1	< 0.001
Female	989,125 (51.0)	738,600 (49.1)	250,525 (57.4)	< 0.001
**Race/Ethnicity**				<0.001
White	1,324,619 (70.4)	1,033,910 (70.9)	290,710 (68.7)	
Black	345,215 (18.4)	255,555 (17.5)	89,660 (21.2)	
Hispanic	129,930 (6.9)	100,240 (6.9)	29,690 (7.0)	
Asian	25,565 (1.4)	23,060 (1.6)	2,505 (0.6)	
Native American	9,070 (0.5)	7,060 (0.5)	2,010 (0.5)	
Other	46,365 (2.5)	37,710 (2.6)	8,655 (2.0)	
**Primary payer**				< 0.001
Medicare	1,015,300 (52.4)	822,585 (54.8)	192,715 (44.2)	
Medicaid	274,485 (14.2)	206,540 (13.8)	67,945 (15.6)	
Private	509,595 (26.3)	365,960 (24.4)	143,635 (32.9)	
Self-pay	77,750 (4.0)	59,320 (4.0)	18,430 (4.2)	
No charge	6,830 (0.4)	5,250 (0.3)	1,580 (0.4)	
Others	53,425 (2.8)	41,760 (2.8)	11,665 (2.7)	
**Median household income**				< 0.001
Quartile 1	565,290 (29.7)	433,615 (29.4)	131,675 (30.7)	
Quartile 2	506,365 (26.6)	388,185 (26.3)	118,180 (27.5)	
Quartile 3	456,365 (24.0)	351,985 (23.8)	104,380 (24.3)	
Quartile 4	377,465 (19.8)	302,155 (20.5)	753,10 (17.5)	
**Hospital Bed Size**				< 0.001
Small	383,044 (19.7)	294,035 (19.6)	89,010 (20.4)	
Medium	552,460 (28.5)	425,150 (28.3)	127,310 (29.2)	
Large	1,004,790 (51.8)	784,605 (52.2)	220,185 (50.4)	
**Teaching status**				0.6426
Non-teaching	552,540 (28.5)	427,850 (28.5)	124,690 (28.6)	
Teaching	1,387,755 (71.5)	1,075,940 (71.5)	311,815 (71.4)	
**Comorbidities**				
Coronary Artery Disease	465,910 (24.0)	363,635 (24.2)	102,275 (23.4)	< 0.001
Congestive Heart Failure	428,920 (22.1)	319,345 (21.2)	109,575 (25.1)	< 0.001
Atrial fibrillation/ flutter	309,765 (16.0)	245,875 (16.4)	63,890 (14.6)	< 0.001
Peripheral Vascular Disease	145,675 (7.5)	117,855 (7.8)	27,820 (6.4)	< 0.001
Hypertension	1,183,720 (61.0)	875,635 (58.2)	308,085 (70.6)	< 0.001
Diabetes Mellitus	485,070 (25.0)	322,665 (21.5)	162,405 (37.2)	< 0.001
Chronic Kidney Disease	291,965 (15.0)	221,080 (14.7)	70,885 (16.2)	< 0.001
Chronic Obstructive Pulmonary Disease	374,725 (19.3)	294,395 (19.6)	80,330 (18.4)	< 0.001
Liver disease	130,505 (6.7)	102,585 (6.8)	27,920 (6.4)	< 0.001
Rheumatological disease	75,275 (0.39)	57,665 (3.8)	17,610 (0.4)	0.0083
Deep Vein Thrombosis	645,065 (33.2)	488,605 (32.5)	156,460 (35.8)	< 0.001

### Trend of obesity in patients with PE over 5 years

Over a period of 5 years, the prevalence of obesity among PE patients increased from 21.5% in 2016 to 24.3% in 2020.

### Incidence of in-hospital therapeutics and association of obesity with in-hospital complications

After adjusting for demographic factors, comorbidities, and reperfusion therapies, a multivariable logistic regression analysis demonstrated that obesity was associated with significantly lower odds of in-hospital mortality (OR, 0.63 [95% CI, 0.60–0.65]; *p* < 0.001), shock requiring vasopressor use (OR, 0.75 [95% CI, 0.71–0.80]; *p* < 0.001) and MIV (OR, 0.78 [95% CI, 0.76–0.81]; *p* < 0.001).

Conversely, there was higher odds of receiving invasive treatment in the obesity cohort, including systemic tPA (OR, 1.59 [95% CI, 1.52–1.66]; *p* < 0.001), surgical thrombectomy/embolectomy (OR, 1.38 [95% CI, 1.15–1.65]; *p* < 0.001), catheter-directed thrombectomy/thrombolysis (OR, 2.23 [95% CI, 2.11–2.36]; *p* < 0.001), and mechanical non-invasive ventilation (NIV) (OR, 1.91 [95% CI, 1.84–1.99]; *p* < 0.001), as detailed in Table 2.

An unadjusted logistic regression analysis also validated these above findings, with obese PE patients having a higher rate of receiving invasive therapies. Obesity was also associated with decreased odds of negative outcomes, including mortality, need for MIV or shock requiring vasopressor use, as indicated in Table 2.

**Table 2:** Adjusted and unadjusted odds ratios of complications and treatments in PE patients with and without obesity (*table view*)

Complication/ Treatment	OR	95% CI	*P*-Value
Mortality	0.63	0.60–0.65	< 0.001
Shock Requiring Pressor Use	0.75	0.71–0.80	< 0.001
Systemic tPA	1.59	1.52–1.66	< 0.001
Surgical Thrombectomy/Embolectomy	1.38	1.15–1.65	< 0.001
Catheter-Directed Thrombectomy/Thrombolysis	2.23	2.11–2.36	< 0.001
MIV	0.78	0.76–0.81	< 0.001
NIV	1.91	1.84–1.99	< 0.001
*Adjusted for age, gender, race, comorbidities (coronary artery disease, congestive heart failure, atrial fibrillation, peripheral vascular disease, hypertension, diabetes mellitus, chronic kidney disease, chronic obstructive pulmonary disease, liver disease, rheumatological disease)			
**Unadjusted OR of Obesity on Treatment and Outcome**			
**Treatment**	**OR**	**95% CI**	**P-Value**
Systemic tPA	1.67	1.60–1.74	< 0.001
Surgical Thrombectomy/Embolectomy	1.55	1.31–1.83	< 0.001
Catheter-Directed Thrombectomy/Thrombolysis	2.26	2.14–2.38	< 0.001
**Outcome**			
Mortality	0.58	0.56–0.61	< 0.001
Mechanical Invasive Ventilation (MIV)	0.85	0.83–0.88	< 0.001
Mechanical Non-Invasive Ventilation (NIV)	1.91	1.84–1.98	< 0.001
Shock Requiring Pressor Use	0.8	0.76–0.85	< 0.001

### Association of obesity with requirements of ventilatory support in pulmonary embolism patients

NIV rates between obese and non-obese patients were significantly different, with almost double the NIV rate of NIV in obese patients (6.12%) compared to non-obese patients (3.30%). Although the rates of NIV were relatively low overall for all PE patients, the difference between the two groups was statistically significant.

## Discussion

Our analysis highlighted a notable increase in the prevalence of obesity among PE patients, increasing from 21.5% in 2016 to 24.3% in 2020. This trend underscores an escalating public health challenge and the need for targeted mitigation strategies against the obesity epidemic in the US [14, 15]. Our findings emphasize the importance of understanding the impact of obesity on PE patients with the hope that studies such as these can be extrapolated into other conditions, providing a comprehensive understanding of the obesity paradox.

In our study, obese PE patients were younger, female, and African American when compared to the demographics of their non-obese counterparts. This suggests a complex interplay of genetic, environmental, and socioeconomic factors that contribute to both obesity and PE risk. Therapeutically, obese patients were more likely to receive advanced therapeutic interventions, including systemic thrombolytics, surgical thrombectomy, and catheter-directed therapies. Despite the higher prevalence of comorbid conditions and increased use of invasive treatments, obese PE patients had lower rates of in-hospital mortality and development of severe complications such as requiring mechanical ventilation or vasopressor support. This “obesity paradox” aligns with recent literature indicating improved survival outcomes for obese individuals compared to non-obese patients who have comorbid cardiovascular and respiratory conditions [6].

Our study found a reduction in the likelihood of in-hospital mortality for obese patients with PE, with an adjusted odds ratio of 0.63 (95% CI 0.61–0.65, *p* < 0.001), even after controlling for demographics, comorbidities and reperfusion therapies. There may be a protective effect of obesity in the context of PE morbidity and mortality, warranting further investigation into the mechanisms behind this phenomenon.

Several protective pathophysiological mechanisms have been proposed to support the enhanced survival among critically ill patients, particularly those with obesity. For obese patients, increased nutritional reserves [5, 16–18] and neurohormonal pathways involved elevated leptin levels [5, 19–21] may confer a survival advantage. We hypothesize that similar protective mechanisms may benefit patients with stroke as acute hospitalizations can impose significant metabolic stress. Predictably, patients with a higher (BMI in our cohort exhibited a high prevalence of cardiovascular risk factors such as hypertension (HTN) and diabetes mellitus (DM). However, it appears that the adverse impact of these cardiovascular risk factors on clinical outcomes may be mitigated by protective mechanisms, potentially improving survival among obese patients hospitalized with PE.

The association between obesity and improved survival after a stroke should not be viewed as a reason to encourage weight gain. Elevated BMI is an established independent risk factor for several cardiovascular conditions, including ischemic heart disease, myocardial infarction, heart failure, atrial and ventricular arrhythmias, sudden cardiac arrest, and stroke [5]. The evidence underscores the importance of preventive strategies to combat obesity, and our findings should not be construed otherwise.

## Limitations

This study has several limitations that warrant consideration while interpreting its findings. First, the potential for information bias arises from the study’s observational design and reliance on discharge data, which may omit critical clinical data such as severity of comorbid conditions or rationales for therapeutic interventions. Minor comorbidities with low severity are less likely to impact outcomes, but the lack of severity differentiation could affect the overall analysis of PE risk factors. We rely on clinical coding standards aligning with diagnostic and imaging standards. Additionally, the NIS database lacks data on long-term outcomes, which restricts our ability to evaluate the persistence of any in-hospital survival advantages observed. We lacked precise timing information regarding the initiation of interventions such as MIV or vasopressor support, preventing us from distinguishing whether these complications functioned as markers of initial disease severity or subsequent clinical outcomes. Certain correlations were difficult to assess directly, thus hindering the extrapolation of findings to clinical applications. Second, the use of ICD-10 codes to identify PE and comorbid conditions may introduce bias. Given the design of the NIS database, the severity of PE (e.g., acute, subacute, massive) could not be categorized based on any validated severity tools (e.g., Pulmonary Embolism Severity Index (PESI)). Finally, we were unable to determine whether non-obese patients represented a potentially sicker cohort with additional risk factors for the development of PE such as immobility. Although we adjusted for multiple confounding variables, residual confounding cannot be fully excluded.

Additionally, immobility plays a crucial role in the outcomes especially among high-risk groups, since it is a significant factor associated with PE risk, but it cannot be assessed in this administrative dataset. Another limitation is the inability to assess the severity of certain comorbid conditions, such as COPD and cardiac failure.

## Clinical implications and future research

The findings from this study carry significant clinical implications. Healthcare providers should be aware of a potential protective effect from obesity in PE patients. However, clinicians should also consider the U-shaped distribution to this effect, where both underweight and extreme obesity may not confer the same benefits, limiting the application of these findings in clinical practice for individuals at the extremes of weight.

Our findings also prompt clinical questions regarding the mechanisms underlying this phenomenon. Future mechanistic studies are essential to elucidate the “obesity paradox” and determine whether the observed outcomes represent a true protective effect of obesity, a “lean paradox,” or are primarily the result of confounding factors [22].

This analysis aligns with the goals of precision medicine, underscoring the need for future studies to explore the biological and physiological factors contributing to the obesity paradox in PE patients, with the potential to advance personalized care strategies [18, 19]. Additionally, future research should compare mortality rates between obese and non-obese individuals receiving various PE interventions, such as systemic thrombolysis, surgical thrombectomy, and catheter-directed thrombolysis. These comparisons could help clarify the factors influencing outcome differences and whether obese and non-obese patients experience similar benefits from therapeutic interventions. Prospective studies employing severity-matching methodologies are encouraged to further investigate these distinctions.

## Conclusion

This study highlights a paradoxical survival advantage in obese patients with PE, despite their higher baseline comorbidities and greater likelihood of requiring advanced therapies. These findings highlight the need for further mechanistic and prospective studies to better understand the complex interplay between obesity, PE and patient outcomes. Such research will be essential for guiding clinical practice and improving patient care. While our large nationwide sample supports the observation of a survival benefit in obese patients with PE, future studies should investigate whether this advantage persists across different obesity classes and explore the heterogeneity within the obesity population. A more refined understanding of these dynamics could lead to more personalized treatment strategies and improved management in PE.

## Data Availability

The data supporting the findings of this study are derived from the National Inpatient Sample (NIS), part of the Healthcare Cost and Utilization Project (HCUP) by the Agency for Healthcare Research and Quality (AHRQ). Access to the NIS requires a data use agreement and can be requested through the HCUP Central Distributor (https://www.hcup-us.ahrq.gov/). As the data are de-identified and publicly accessible, this study was deemed exempt from Institutional Review Board (IRB) review per federal regulations.
